# Risk-predicted dual nomograms consisting of clinical and ultrasound factors for downgrading BI-RADS category 4a breast lesions - A multiple centre study

**DOI:** 10.7150/jca.51302

**Published:** 2021-01-01

**Authors:** Zihan Niu, Jia-Wei Tian, Hai-Tao Ran, Wei-Dong Ren, Cai Chang, Jian-Jun Yuan, Chun-Song Kang, You-Bin Deng, Hui Wang, Bao-Ming Luo, Sheng-Lan Guo, Qi Zhou, En-Sheng Xue, Wei-Wei Zhan, Qing Zhou, Jie Li, Ping Zhou, Chun-Quan Zhang, Man Chen, Ying Gu, Jin-Feng Xu, Wu Chen, Yu-Hong Zhang, Hong-Qiao Wang, Jian-Chu Li, Hong-Yan Wang, Yu-Xin Jiang

**Affiliations:** 1 Department of Ultrasound, Peking Union Medical College Hospital, Chinese Academy of Medical Sciences and Peking Union Medical College, Beijing 100730, China.; 2 Department of Ultrasound, the Second Affiliated Hospital of Harbin Medical University, Harbin 150086, China.; 3 Department of Ultrasound, the Second Affiliated Hospital of Chongqing Medical University, Chongqing 400010, China; Chongqing Key Laboratory of Ultrasound Molecular Imaging, Chongqing 400010, China.; 4 Department of Ultrasound, Shengjing Hospital of China Medical University, Shenyang 110004, China.; 5 Department of Medical Ultrasound, Fudan University Shanghai Cancer Center & Department of Oncology, Shanghai Medical College, Fudan University, Shanghai 200032, China.; 6 Department of Ultrasonography, Henan Provincial People′s Hospital, Zhengzhou 450003, China.; 7 Department of Ultrasound, Shanxi Academy of Medical Science, Dayi Hospital of Shanxi Medical University, Taiyuan 030032, China.; 8 Department of Medical Ultrasound, Tongji Hospital, Tongji Medical College of Huazhong University of Science and Technology, Wuhan 430030, China.; 9 Department of Ultrasound, China-Japan Union Hospital of Jilin University, Changchun 130033, China.; 10 Department of Ultrasound, the Sun Yat-sen Memorial Hospital, Sun Yat-sen University, Guangzhou 510120, China.; 11 Department of Ultrasonography, First Affiliated Hospital of Guangxi Medical University, Nanning 530021, China.; 12 Department of Medical Ultrasound, the Second Affiliated Hospital, School of Medicine, Xi'an Jiaotong University, Xi'an 710004, China.; 13 Department of Ultrasound, Union Hospital of Fujian Medical University, Fujian Institute of Ultrasound Medicine, Fuzhou 350001, China.; 14 Department of Ultrasound, Ruijin Hospital, Shanghai Jiaotong University, School of Medicine, Shanghai 200025, China.; 15 Department of Ultrasonography, Renmin Hospital of Wuhan University, Wuhan 430060, China.; 16 Department of Ultrasound, Qilu Hospital, Shandong University, Jinan 250012, China.; 17 Department of Ultrasound, the Third Xiangya Hospital of Central South University, Changsha 410013, China.; 18 Department of Ultrasound, the Second Affiliated Hospital of Nanchang University, Nanchang, Jiangxi, China.; 19 Department of Ultrasound Medicine, Tongren Hospital, Shanghai Jiao Tong University School of Medicine, Shanghai 200336, China.; 20 Department of Ultrasonography, the Affiliated Hospital of Guizhou Medical University, Guiyang 550004, China.; 21 Department of Ultrasound, Shenzhen People's Hospital, the Second Clinical Medical College of Jinan University, Shenzhen 518020, China.; 22 Department of Ultrasound, the First Hospital of Shanxi Medical University, Taiyuan 030001, China.; 23 Department of Ultrasound, the Second Hospital of Dalian Medical University, Dalian 116027, China.; 24 Department of Ultrasound, the Affiliated Hospital of Qingdao University, Qingdao 266003, China.

**Keywords:** Breast cancer, ultrasonography, risk factors, elastography, Nomogram

## Abstract

**Purpose:** To develop and to validate a risk-predicted nomogram for downgrading Breast Imaging Reporting and Data System (BI-RADS) category 4a breast lesions.

**Patients and Methods:** We enrolled 680 patients with breast lesions that were diagnosed as BI-RADS category 4a by conventional ultrasound from December 2018 to June 2019. All 4a lesions were randomly divided into development and validation groups at the ratio of 3:1. In the development group consisting of 499 cases, the multiple clinical and ultrasound predicted factors were extracted, and dual-predicted nomograms were constructed by multivariable logistic regression analysis, named clinical nomogram and ultrasound nomogram, respectively. Patients were twice classified as either “high risk” or “low risk” in the two nomograms. The performance of these dual nomograms was assessed by an independent validation group of 181 cases. Receiver Operating Characteristic (ROC) curve and diagnostic value were calculated to evaluate the applicability of the new model.

**Results:** After multiple logistic regression analysis, the clinical nomogram included 2 predictors: age and the first-degree family members with breast cancer. The area under the curve (AUC) value for the clinical nomogram was 0.661 and 0.712 for the development and validation groups, respectively. The ultrasound nomogram included 3 independent predictors (margins, calcification and strain ratio), and the AUC value in this nomogram was 0.782 and 0.747 in the development and validation groups, respectively. In the development group of 499 patients, approximately 50.90% (254/499) of patients were twice classified “low risk”, with a malignancy rate of 1.18%. In the validation group of 181 patients, approximately 47.51% (86/181) of patients had been twice classified as “low risk”, with a malignancy rate of 1.16%.

**Conclusions:** A dual-predicted nomogram incorporating clinical factors and imaging characteristics is an applicable model for downgrading the low-risk lesions in BI-RADS category 4a and shows good stability and accuracy, which is useful for decreasing the rate of invasive examinations and surgery.

## Introduction

According to global cancer statistics in 2018, breast cancer is the most common cancer in women and is the leading cause of cancer deaths (15.0% of all cancer deaths in women) 1. Ultrasound, as a radiation-free and non-invasive method, is the preferred approach for breast examination, especially for dense breasts 2-3. According to the American College of Radiology (ACR) Breast Imaging Reporting and Data System (BI-RADS) 4-5, breast lesions are divided into 6 categories based on different ultrasonic characteristics. Category 4 lesions have great malignant probability, varying from 2% to 95%, which are further classified into the 4a, 4b, and 4c subcategories and suggested for further examination, such as puncture biopsy or surgical treatment. The malignancy rate of type 4a lesions is only 2% to 10%, and most of them are benign, which leads to the low specificity in ultrasound. If risk prediction is performed for grade 4a lesions and low-risk lesions were conducted follow-up observation, unnecessary invasive examinations could be reduced.

The issue of how to develop a simple and effective breast cancer risk prognostic model has become the focus of breast cancer prevention. The Breast Cancer Risk Assessment Tool (BCRAT) 6, also known as the Gail Model, which is based on American Caucasian data, was proposed in 1989 by Costantito et al., through many studies and years of testing and corrections, the Gail Model has been the most widely used and is one of the standard methods for breast cancer risk assessment, especially in European and American countries. Several risk-predicted models for breast cancer have been reported with different risk factors 7-10. Because of disparities in the various racial and ethnic groups, the application of the Gail Model has certain limitations in the Asian population 11-13. If a 4a lesion risk-prediction model can be established based on Asian population data, the patients can be divided into "high-risk” and "low-risk” populations according to the clinical risk degree results, which will help to further improve the diagnostic accuracy and to avoid missed diagnoses.

According to BI-RADS, conventional ultrasound (US), including two-dimension (2D) and colour Doppler, could provide information for discrimination of breast lesions 14,15. However, there are overlapping morphological features in some benign and malignant lesions. Elastography as an add-on to the conventional imaging is non-invasive and enables quantitatively assessing the tissue stiffness objectively and has been used in many diseases. In addition, elastography was added to BI-RADS in the new version in 2013, and its diagnostic value was confirmed. Based on previous studies, tissue stiffness is associated with the risk of malignancy; the harder the lesion is, the greater the probability of malignant risk is 16-18. Ultrasound is increasingly used in clinical breast examination and has demonstrated good performance not only in breast tumour differential diagnoses but also for the potential to downgrade BI-RADS 4a lesions to reduce false-positive biopsies without increasing the risk of missing cancers 19.

Most of the previous literature used clinical factors or ultrasound signs alone to distinguish benign and malignant breast nodules. According to the study by Jieun Koh et al., BI-RADS 4a lesions were classified into “average” and “high” risk by personal or family history, and “soft” and “not soft” by elastography. Only the lesions with “average risk” and “soft stiffness” could be downgraded without further examination 19. It was found that the missed diagnosis rate for malignant lesions was only 1.5%, and 26.7% (68/255), respectively, and benign nodules could be degraded. To evaluate clinical risk factors more comprehensively, to identify suspicious signs of traditional ultrasound and to analyse elastography quantitatively, this study established a risk factor-predicted model for downgrading 4a breast lesions, which is more suitable for the Chinese population and to reduce the rate of unnecessary examinations and surgery.

## Materials and Methods

This was a multi-centre study conducted at regional medical centres in China, including 32 hospitals from 23 different provinces. All hospitals and participating radiologists completed real-name registration on the website (www.nuqcc.cn) and uploaded information after approval. To reduce the difference in diagnosis between sonographers and to improve the proficiency and consistency, before the multi-centre research, the doctors of each centre had been trained through on-site operation, demonstration and practice. All the data and images from the website were separately reviewed by three experienced radiologists in our hospitals. When there was a discrepancy, the consensus was reached after discussion. This study was registered at Chinese Clinical Trail Registry platform (http://www.chictr.org.cn) with an approval number of ChiCTR1900023916. Informed consent was obtained from all individual participants included in the study.

### Study population

In total, 708 lesions diagnosed as 4a were selected from 3020 consecutive breast cancer patients who underwent biopsy and surgery and who were diagnosed by ultrasound from December 2018 to June 2019 in 32 hospitals.

Inclusion criteria were as follows: (1) patients who underwent breast lesion elastography examination; (2) patients with available pathological results; and (3) patients with available clinical information. The exclusion criteria included the following: (1) patients who had a history of preoperative radiotherapy, chemotherapy, or endocrine therapy; (2) patients for whom ultrasound images were not clear; and (3) patients for whom the elastography imaging was not satisfactory, such as patients with a cough who could not cooperate with elastography imaging.

**Figure 1:**
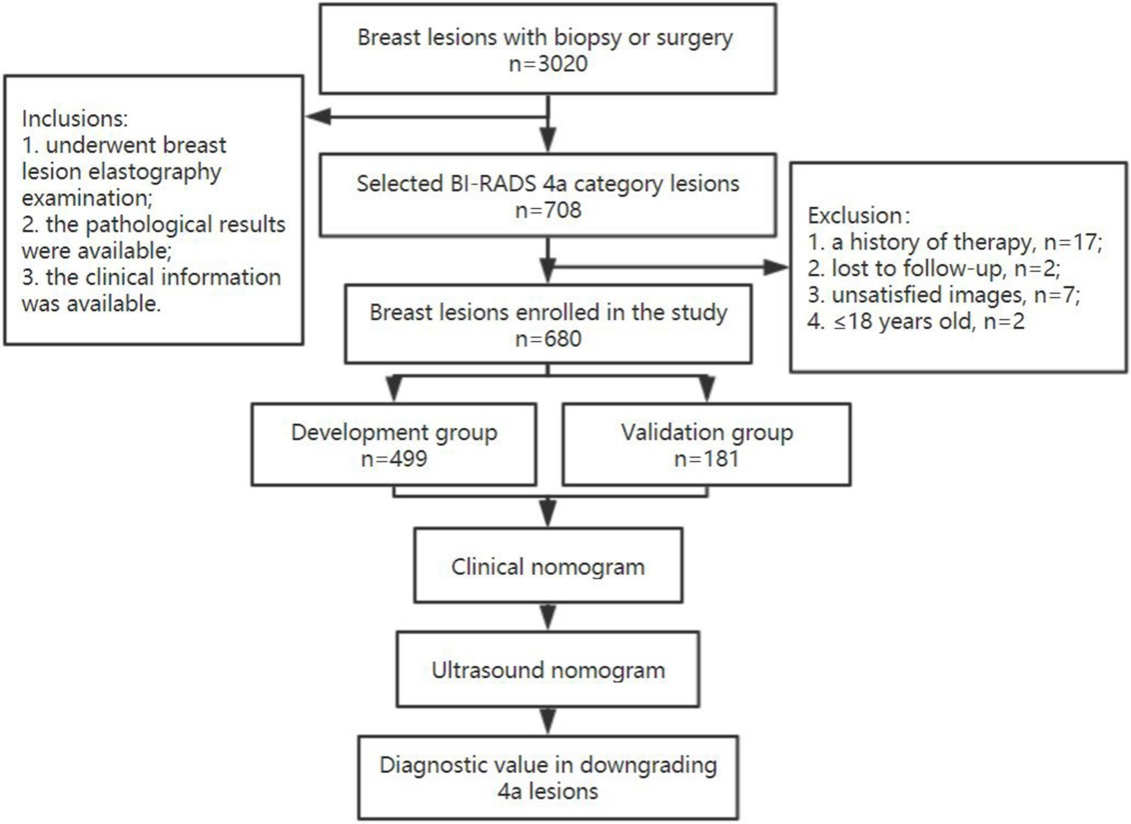
The flow chart of this study.

A total of 680 patients were finally enrolled, including 639 with benign lesions and 41 with malignant lesions. All 4a lesions were randomly divided two groups in a ratio of 3:1 as development and validation groups, respectively. The study flow chart is shown in **Figure 1.** All of these patients underwent breast ultrasound examination prior to core needle biopsy or surgical pathology. The final pathologic results were considered the gold standard.

### Clinical characteristics acquisition

Clinical characteristics based on the Gail Model, including personal history of breast cancer, first-degree relatives with breast cancer history (yes or no), a personal history of atypical ductal hyperplasia (ADH), and height and weight were recorded. In addition, body mass index (BMI) was calculated by the following formula: BMI = weight (kg)/height (m)^2^.

### Ultrasonic imaging acquisition

All US examinations were performed with Resona 7 or 8 devices (Mindray Medical, Shenzhen, China) equipped with 5-14 MHz linear-array transducers. Conventional US and elastography were prospectively recorded before biopsy or surgery within two weeks by 32 sonographers with more than 3 years of experience who were blinded to the patient clinical data. For each patient, the ultrasound images of only one lesion with the highest BI-RADS categories was reserved.

The US examination included 2D and colour Doppler imaging. In 2D ultrasound, the breast tissue constitution (homogeneous background echotexture-fat, homogeneous background echotexture-fibroglandular, heterogeneous background echotexture), maximum diameters, shape (oval, round, irregular), orientation (parallel, not parallel), margin (circumscribed; one uncircumscribed feature, including indistinct, angular, microlobulated, and spiculated; more than two uncircumscribed features), echo pattern (anechoic, hyperechoic, complex cystic and solid, hypoechoic, isoechoic, heterogeneous), posterior features (no posterior features, enhancement, shadowing, combined pattern), calcification position (calcification in a mass; calcification outside of a mass; intraductal calcification), calcification morphology (micro calcification, coarse calcification), associated features (architectural distortion, duct discharges, skin changes, oedema) were collected. In Doppler ultrasound, the vascularity (absent, internal, peripheral) and the amount of blood signals (grades 0, 1, 2, 3) based on Adler's index were assessed 20. Each lesion was categorized according to the possible malignancy of US BI-RADS (American College of Radiology, 2013) when there were one or more suspicious malignant findings, such as round or irregular shape, uncircumscribed margin, non-parallel orientation, associated features, calcification, complex echogenicity, and posterior shadowing.

The elastography model was switched after ascertaining that the lesion was in the largest diameter section and the B-mode images were optimal. To acquire reliable results, the angle of the probe was kept perpendicular to the skin and appropriate manual compression in the normal range was applied to keep the colour of the entire target stable. A wide colour spectrum of red to green to blue was displayed in elastography images, representing tissue from hard to intermediate to soft component. When the fatty tissue on the surface of the mass was blue, the elastography image was saved. Based on the colour, a Tsukuba score from 1 to 5 was assigned 21. Scores from 1 to 5 indicated a uniform soft strain in the entire hypoechoic lesion, a mixed pattern, hard but smaller on elastogram, the same size on elastogram, and hard and larger on elastogram than in 2D images, respectively. Strain ratio was measured and recorded through drawing the region of interest location (ROI). The tumour ROI was placed entirely in the tumour, and the subcutaneous fat ROI was limited to fat not containing fibroglandular breast tissue at a similar depth to the lesion. The elastography imaging was displayed twice for every lesion and the average strain ratio was recorded.

### Data and statistical analysis

Continuous variables are expressed as the mean±SD and were tested with Kruskal-Wallis rank sum test. Categorical variables are presented as frequencies and percentages and were analyzed by chi-square test. Variables showing *P* < 0.05 in univariate analysis were considered possible predictors and were entered in the multivariate model. Two nomograms, clinical nomogram and ultrasound nomogram, were built in the training cohort based on multivariate analysis.

Based on the significant clinical predicted factors in the development group, the patients were categorized as “clinical high risk” and “clinical low risk”. In addition, based on the ultrasonic predicted factors in the development group, the patients were divided as “ultrasound high risk” and “ultrasound low risk”.

The pathological diagnosis was used as the “gold standard”, and the area under the receiver operating characteristic (ROC) curve (AUC) was calculated after determining a cut-off value by analyzing the nomogram. The Hosmer-Lemeshow (HL) test was assessed to evaluate the calibration. TP, TN, FP, and FN represented the number of true-positive findings, true-negative findings, false-positive findings and false-negative findings, respectively.

Then, the dual nomograms built in the development group were further verified in the validation cohort. The performance of the model in terms of discrimination and diagnostic value was assessed in the validation cohort using the same methods described above.

The software SPSS Statistics (version 24.0, USA) and R software (version 3.3.0) were used for data analysis. A *P* value of <0.05 was considered significantly different.

## Results

### Clinical and pathological patient characteristics

The development group was comprised of 499 cases (473 benign lesions and 26 malignant lesions; mean age 42.13±10.85). An independent validation group included 181 cases (166 benign lesions and 15 malignant lesions; mean age 42.52±11.84). Histopathological diagnoses of the 680 breast masses were confirmed via US-guided core needle biopsy of 107 lesions and surgery of 573 lesions.

The pathology results in the development and validation cohorts are summarized in **Table 1.** The malignant lesion rate was 5.21% (26/499) and 8.29% (15/181) in the training and validation cohorts, respectively. The malignant lesions included ductal carcinoma *in situ* (DCIS), invasive ductal cancer (IDC), and invasive lobular cancer (ILC) mucinous carcinoma (MC) and invasive cancer (IC). In addition, fibroadenoma, adenosis and intraductal papilloma were the most common benign lesions.

### Clinical predicted nomogram

Base on the Gail Model and previous study, clinical characteristics, including mean age, BMI, family history of first-degree relatives with breast cancer history, age at menarche, number of births, age at first birth and ADH history, were assessed as risk factors. The results showed that only mean age and family history were significant in in both the development and validation groups (**Table 2**).

**Figure 2:**
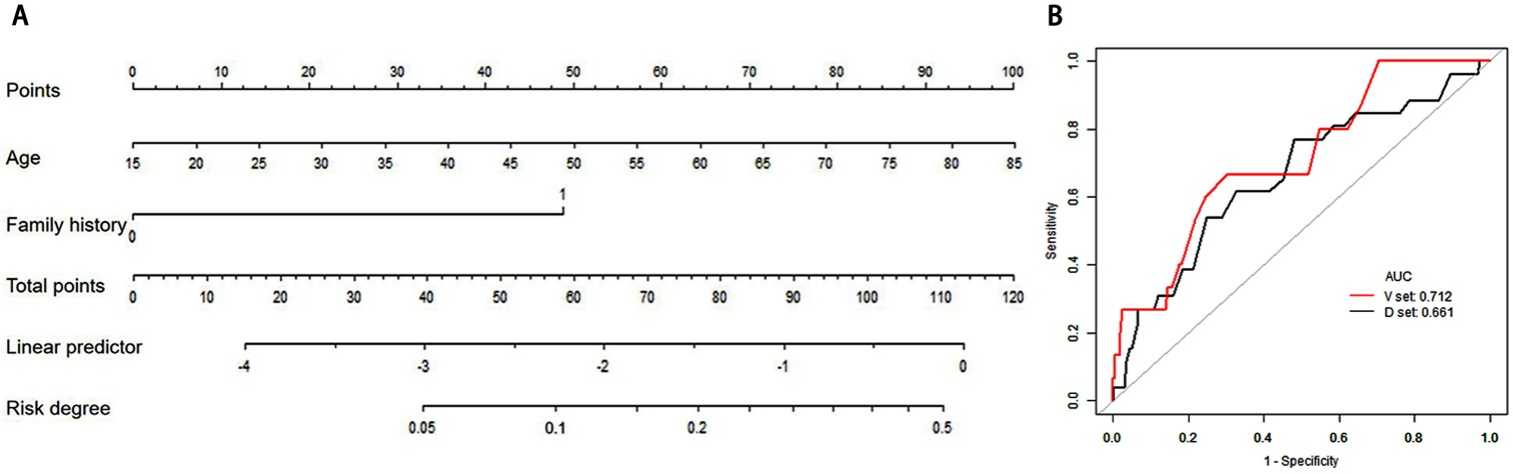
** Clinical nomogram and its diagnostic value. A.** Nomogram for assessing the risk degree based on clinical factors. To use this nomogram, first locate the patient's age, then draw a straight line up to the Points axis at the top to get the score associated with age. Repeat the process for the family history. Add the score of both covariates together and locate the total score on the Total points axis. Next, draw a line straight down to the “risk degree” axis at the bottom to obtain the probability. B. Receiver operating characteristic (ROC) curve in clinical risk predicted model in both develop and validation cohorts. Area under the curve (AUC) in the development set was 0.661 (Black line), and AUC in the validation set was 0.712 (Red line).

The variables in **Table 1** were assessed in a univariate logistic regression analysis, and the variables with outcomes of P < 0.05 were entered into a multivariate logistic regression (**Table 3**). The results showed that age (OR =1.05, 95% CIs: 1.03 to 1.08) was an independent predictor. Many previous studies have shown that family history is strongly associated with breast cancer 22-24, even in multivariate regression, family history (OR =7.13, 95% CIs: 0.62 to 82.03) with *P=*0.1125, the number of first-degree relatives with history of breast cancer was an independent predictor and was forced to be incorporated into the logistic regression analysis. The formula of Linear Predictor = -4.78455 +0.04167*Age +1.96384*(family history=1). A model incorporating these two independent predictive factors was built and is shown as a nomogram (**Figure 2A**). To use the nomogram, first, the subject's age and family history can be located on the relevant axis. Next, a straight line is drawn upwards, to the point of the axis on the top, to acquire the points received based on covariates, respectively. Total points are calculated by adding all the points obtained from every covariate. The final sum is located on the total points axis, and a straight line was drawn downwards from there to obtain the probability of risk degree. Through the nomogram, the cut-off of risk degree was 0.0593. These patients were regarded as “clinical high risk” with risk degree more than or equal to 0.0593, and as “clinical low risk” with risk degree less than 0.0593.

In internal validation, the ROC showed the resulting model with an AUC of 0.661. The Hosmer-Lemeshow test was not significant (P = 0.694), suggesting a good fit of the model. In the independent validation cohort, the clinical model displayed moderate discrimination with an AUC of 0.712 (**Figure 2B**). Moreover, the Hosmer-Lemeshow test (P =0.358 ) was not significant. The diagnostic value of the clinical nomogram in the development and validation groups is shown in **Table 4.** In the development group, the sensitivity, specificity and accuracy were 0.5385, 0.7526 and 0.7415, respectively. With the same cut-off as the development group, the sensitivity, specificity and accuracy in the validation group were 0.5333, 0.7831 and 0.7624, respectively.

### Ultrasound-predicted nomogram

According to BI-RADS, the ultrasound characteristics and their P values are shown in **Table 5.** The results showed that margin, shape, calcification morphology, position and elastography index were significantly different in the development group. In addition to the above parameters, there were other parameters, such as echo pattern and structural distortion, which had certain significant differences in the validation group.

**Figure 3:**
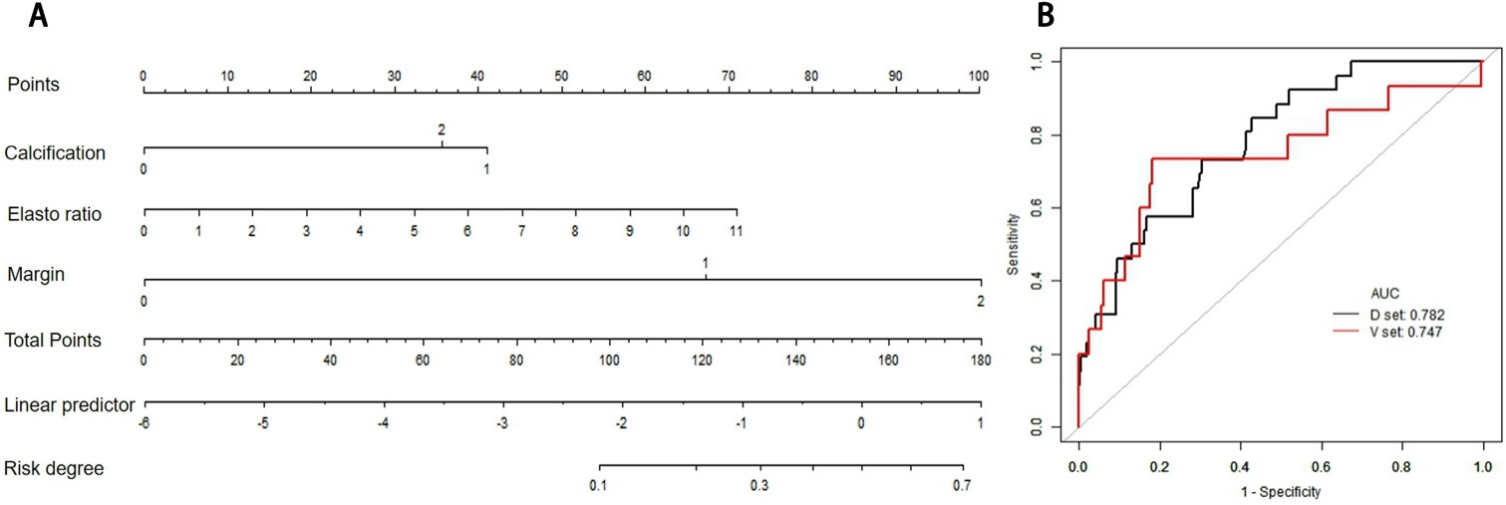
** Ultrasound nomogram and its diagnostic value. A.** Nomogram for assessing the risk degree based on ultrasonic-predicted factors. Draw a straight line up to the Points axis at the top to get the score associated with margin, calcification and strain ratio, and add the score of both covariates together and locate the total score on the Total points axis. Next, draw a line straight down to the “risk degree” axis at the bottom to obtain the probability. **B.** Receiver operating characteristic (ROC) curve for ultrasonic risk predicted model in both the development and validation cohorts. ROC curve of diagnostic performance for malignant lesions with AUC of 0.782 in the development group and 0.747 in the validation cohort.

The variables in **Table 5** were assessed in a univariate logistic regression analysis, and the variables with outcomes of *P* < 0.05 were entered into a multivariate logistic regression (forward stepwise logistic regression) (**Table 3**), where variables with *P* < 0.05 were considered possible predictors. Margin, calcification, and strain ratio were identified as independent predictors of patient classification as “high risk” or “low risk”. The formula of Linear Predictor = -5.46644 +1.44638(Margin=1) +2.50926*(Margin=2) +0.26413*(Calcification=1) +1.68924*(Calcification=2) +0.32118*Strain ratio. A model was built and is shown as a nomogram (**Figure 3A**).

The probability of ultrasound risk degree was obtained from the nomogram in the same method as above. The total points are calculated by adding all the points obtained from margin, calcification morphology, and strain ratio and the cut-off of risk degree was 0.0486. Patients were regarded “ultrasound high risk” with risk degree more than or equal to 0.0486, and “ultrasound low risk” with risk degree less than 0.0486.

In the development and validation cohorts, the discrimination of ultrasound risk nomogram was moderate with AUC of 0.782 (*P =* 0.905) and 0.747 (*P* = 0.359), respectively (**Figure 3B**). In addition, the Hosmer-Lemeshow test was not significant.

### The dual nomogram diagnostic process in the development group

**Figure 4:**
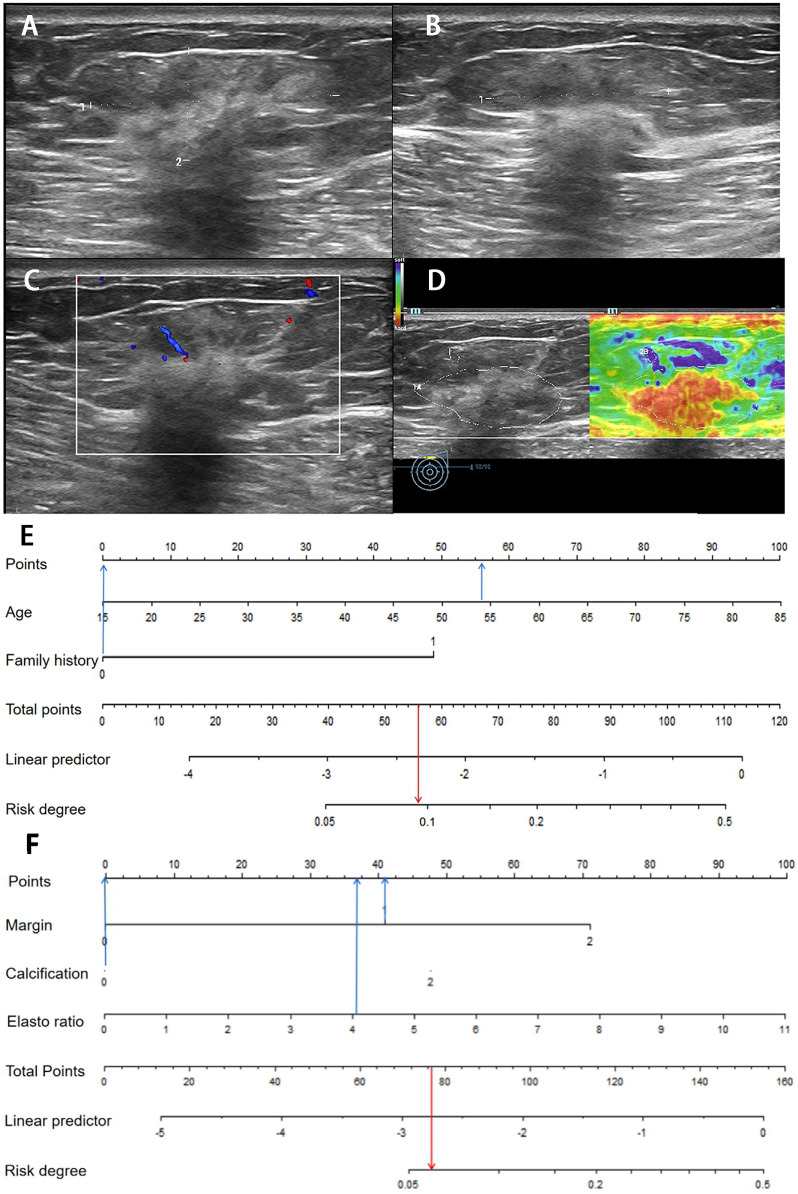
** Images from a 54-year-old woman with a BI-RADS category 4a lesion in the left breast and the pathology was invasive ductal carcinoma.** (**A**) Lesion size was 3.2*1.4 cm on B-mode imaging in the longest axis of the mass. The lesion showed unclear boundary. (**B**) The third measurement of the lesion from a view orthogonal to the first image. (**C**) Grade 1 in Colour Doppler. (**D**) The elastic ratio was 4.13 (blue represents soft and red represents hard). (**E**) In the clinical nomogram, the patient was high risk with 0.0735. (**F**) The lesion was classified as high risk in the ultrasound nomogram with a risk rate of 0.0634.

**Figure 5:**
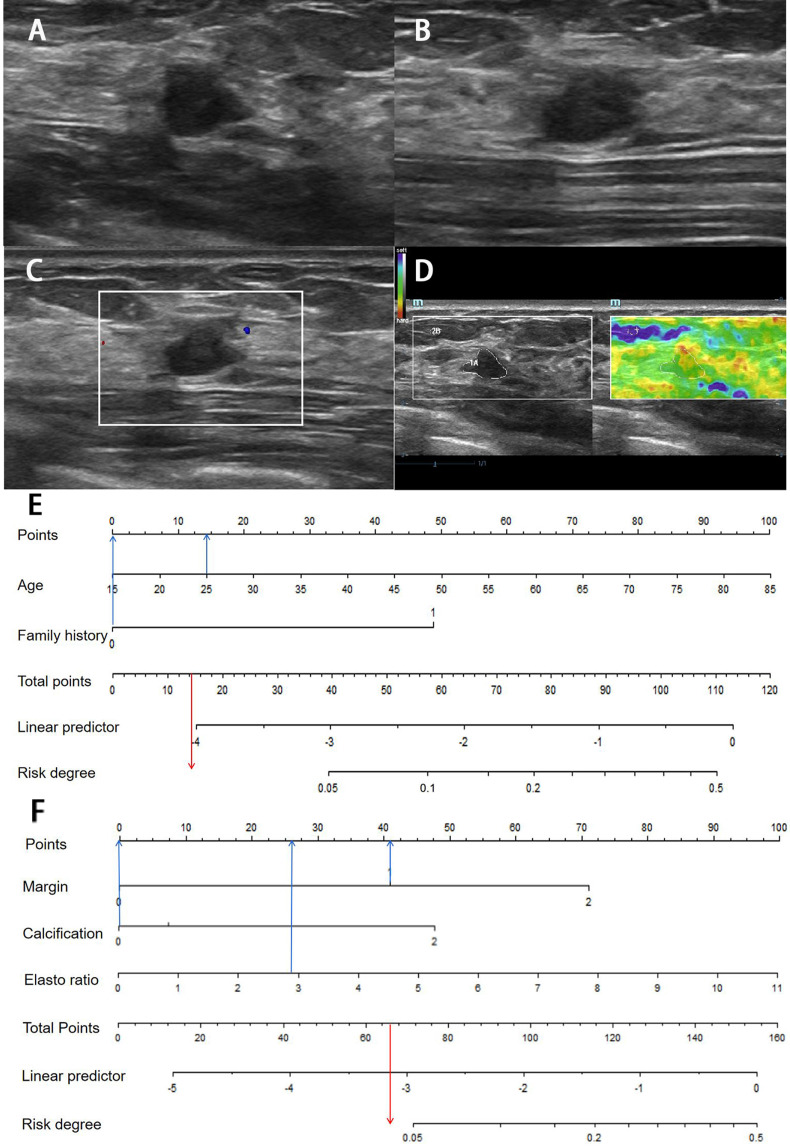
** Image of a fibroadenoma in a 25-year-old woman with a BI-RADS category 4a lesion in the right breast.** (**A**) Lesion size was 0.8*0.6 cm on B-mode imaging in the longest axis of the mass. The lesion showed indistinct margin. (**B**) The third measurement of the lesion from a view orthogonal to the first image is 0.7 cm. (**C**) Grade 0 in Colour Doppler. (**D**) The elastic ratio was 2.89 (blue represents soft and red represents hard). (**E**) In the clinical nomogram, the patient was classified as low risk with 0.0231. (**F**) The lesion was classified as low risk in the ultrasound nomogram with risk degree of 0.0434.

For the dual nomogram established in this research, the internal validation was performed in the development group. First, the patients were divided into high-risk 26.25% (131/499) and low-risk 73.75% (368/499) groups based on clinical nomogram. Then, through the ultrasound nomogram, the patient was given a second risk-degree classification (**Figures 4 & 5**).

**Figure 6:**
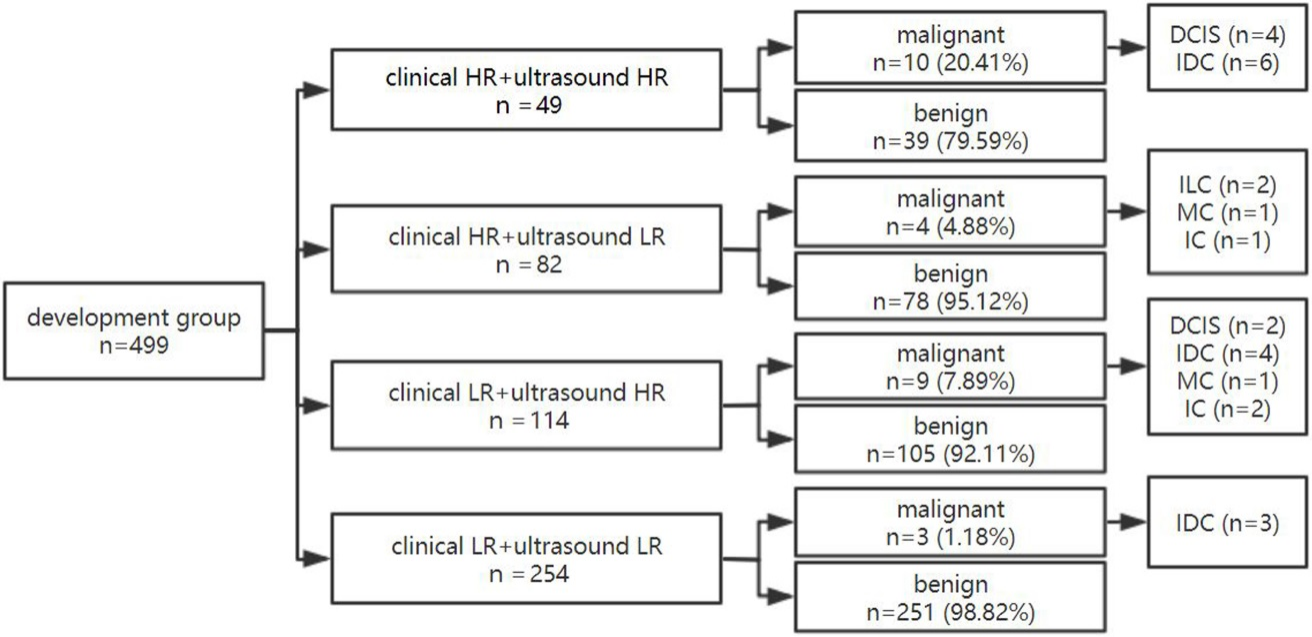
** The diagnostic process of dual nomogram in the development group.** Abbreviations: HR, high risk; LR, low risk.

Of 131 lesions in clinical high-risk women, 37.40% (49/131) of lesions showed high-ultrasonic risk and 62.60% (82/131) showed low risk by ultrasound. The malignancy rate of lesions with dual high risk was 20.41% (10/49). Of 368 lesions in clinical low-risk women, 30.98% (114/368) showed high-ultrasonic risk and 69.02% (254/368) showed low risk by ultrasound. Of 254 lesions with both clinical and ultrasonic low risk, only 3 lesions were malignant and diagnosed as IDC pathologically, of which the malignancy rate was 1.18% and far below those with dual high-risk lesions. When either the clinical or ultrasonic risk was high, the malignancy rate was 4.88% and 7.89%, respectively (**Figure 6**).

### The dual nomogram diagnostic process in the validation group

**Figure 7:**
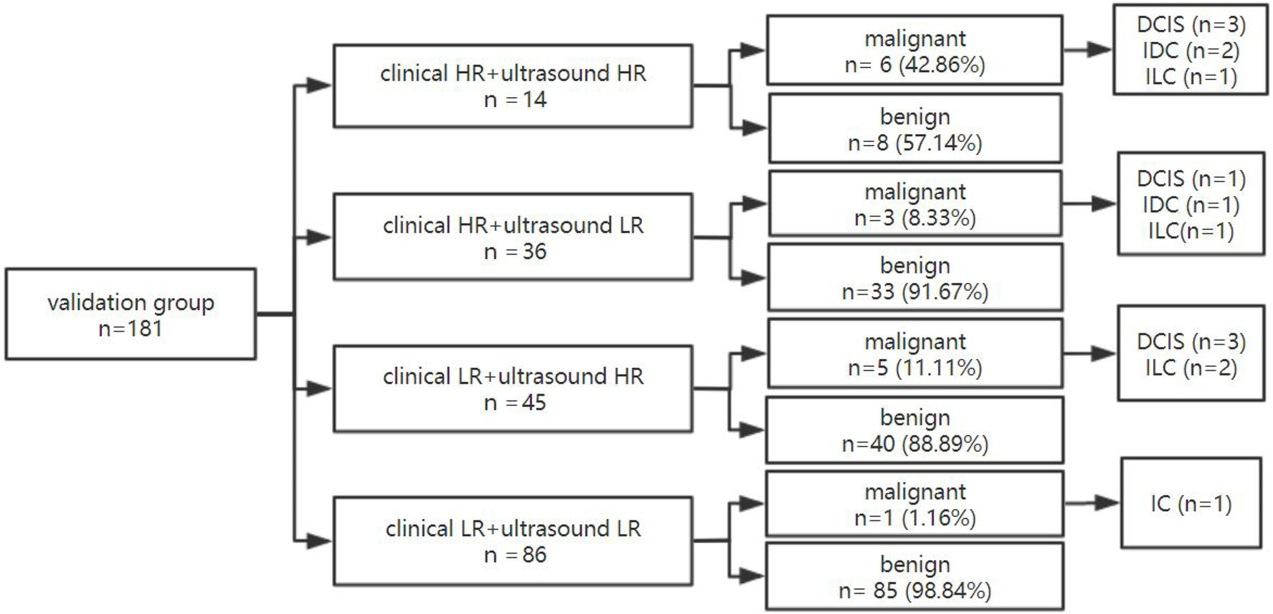
** The diagnostic process of dual nomogram in the validation group.** Abbreviations: HR, high risk; LR, low risk.

In the validation group, 181 lesions, 27.62% (50/181) showed high clinical risk and 72.38% (131/181) low clinical risk (**Figure 7**). The malignancy rate of lesions in women with both ultrasound and clinical low risks was 1.16% (1/86) and was significantly lower than the respective 42.86% (6/14) malignancy rate of lesions in women with both high clinical and ultrasound risks.

When one of the two nomograms was high risk, the malignancy rate could be 8.33% (3/36) and 11.11% (5/45), respectively. Through this model, approximately 47.51% (86/181) of lesions with dual low-risk lesions could be downgraded with a missed diagnosis rate of only 1.16%.

## Discussion

Breast cancer has become a disease of global concern. Category 4a lesions have a malignancy rate of 2% to 10%, with low specificity in ultrasound diagnosis, which leads to unnecessary invasive examination of benign diseases. To establish an efficient model for downgrading 4a lesions, this study constructed dual nomograms based on a Chinese sample, and the patients were twice classified as either “high risk” or “low risk” in clinical and ultrasound nomograms. The diagnostic performance of dual nomograms was validated in another group. The results showed that whether internal or external validation, the model constructed in this study can effectively discriminate 4a lesions.

There are currently multiple breast cancer risk assessment models, and the existing risk prediction models have similar, moderate predictive accuracy overall 25. The Gail Model is the most widely used and is one of the standard methods for breast cancer risk assessment. Moreover, its predicted value has been assessed in previous studies with different results. According to Gao et al., the model containing only age at menarche, age at first birth and number of first-degree relatives with breast cancer could provide a more convenient way to predict the risk of invasive breast cancer in Southeast Asian women 26. Sa-Nguanraksa D conducted a study to evaluate whether the Gail Model can calculate the risk of breast cancer in Thai women and found age, parity, age at first live birth, and history of atypical ductal hyperplasia (ADH) were significant risk factors for breast cancer 27. In our study, the patient's age and family history are two of the most significant variables for patients with 4a lesions, which is similar to previous studies. Other factors, such as ADH and age at first birth, were not associated with breast cancer risk, which may be due to the differences in the sample population enrolled and sample size restrictions.

According to BI-RADS, 4a category lesions have certain malignancy signs, but benign and malignant are not easy to distinguish. The ultrasound findings of some benign and malignant lesions in this study were overlapping. In the development group, 88.46% of malignant nodules grew in parallel, and 23.08% of lesions had clear margins. Furthermore, benign lesions could also show malignant signs; for example, 41.44% had unclear margins and 7.82% were non-parallel. For 4a lesions, selecting the riskiest malignant signs is helpful to distinguish the lesions and to improve the diagnostic value. This study found that margins and calcified morphology were significantly different. According to logistic regression analysis, the OR value of coarse calcification and micro-calcification was 0.98 (*P=*0.9766) and 4.67 (*P=*0.0006), respectively. Micro-calcification was significantly associated with malignant lesions, which is the same as previous studies 28. In addition, the margins in this study were highly significant for the diagnosis of 4a lesions. Unclear margins include indistinct, angular, microlobulated and spiculated, and when more than two cases are combined, the rate of malignancy risk is higher.

In the new version of BI-RADS guidelines, elastography as a new predictor has been added to assess breast lesions and has been reported with a high diagnostic value in the differentiation of breast lesions in many studies. In a meta-analysis of 2087 lesions by 9 studies, Sadigh et al. summarized the accuracy of elastography for differentiation of malignant and benign breast abnormalities and found the pooled sensitivity of 88% and specificity of 83% when using strain ratio, and the pooled sensitivity of 98% and specificity of 72% when using length ratio 29.

Han et al. compared different elastic methods, including strain elastography (SE), acoustic radiation force impulse-inducing Virtual Touch Imaging (VTI), and Virtual Touch Imaging Quantification (VTIQ), in downgrading US BI-RADS category 4a lesions. The authors found that 50.8% to 85.8% of lesions were downgraded with a malignancy rate range from 0% to 50% when using different combinations of elastography methods, which showed the combination of different elastic methods has the potential to downgrade BI-RADS 4a lesions with excellent performance 30. This study added shear elastography on the basis of conventional ultrasound and combined clinical characteristics, and approximately 50.90% in the development group and 47.51% in the validation group 4a lesions with both low risks could be downgraded with the false negative rate of only 1.18% or 1.16%, respectively, which increased diagnostic value for the identification of benign and malignant lesions and showed excellent stability. The malignancy rate of BI-RADS 4a lesions with dual “low risks” is in line with the proportion of BI-RADS 3 lesions (<2%) and could be followed up by this dual nomogram, which is conducive to reducing unnecessary invasive biopsies and saving clinical resources.

There are some limitations in this study. First, only patients with pathology results were enrolled in this study, and some patients in follow-up were not included, which may have led to selection bias and resulted in the underestimation of the NPV and the overestimation of the PPV. Second, the predicted value of breast cancer family history (only 3 in the benign group and 2 in the malignant group with a positive breast cancer family history) may be limited because of the moderate sample size of the study. We used the forced inclusion logistic regression and incorporated “family history” as one risk factor to reduce the deviation of modal.

In this study, we extracted meaningful factors from a Chinese multi-centre sample and established a dual-risk predicted model by clinical and ultrasound factors. The patients who had both low risks could be downgraded and could be followed up with a low malignancy rate, which was less than 2%. In conclusion, this dual nomogram, taking clinical risk factors and ultrasound characteristics together, showed high predictive value for downgrading US BI-RADS 4a lesions in both the development and validation groups, which is useful to reduce invasive examinations and surgery, and can be used as a screening tool for risk stratification among the 4a lesions.

## Figures and Tables

**Table 1:** Pathological diagnosis of 450 BI-RADS category 4a breast lesions

Parameter	Development group		Validation group	
	Benign lesions	Malignant lesions	Benign lesions	Malignant lesions
Total	n=473	n=26	n=166	n=15
Fibroadenoma	n=272		n=97	
Adenosis	n=113#		n=47	
Intraductal papilloma	n=37*		n=10	
Abscess or mastitis	n=18		n=5	
Hyperplasia	n=18		n=3	
Hamartoma	n=1		n=0	
Benign phyllodes tumour	n=3		n=1	
Scar	n=1		n=0	
Other benign diagnoses	n=10		n=3	
Ductal carcinoma *in situ*		n=6		n=7
Invasive ductal cancer		n=13		n=3
Invasive lobular cancer		n=2		n=4
Mucinous carcinoma		n=2		n=0
Invasive cancer		n=3		n=1

*One intraductal papilloma is with focal atypical ductal hyperplasia; #One adenosis is with focal atypical ductal hyperplasia.

**Table 2:** Clinical characteristics of category BI-RADS 4a breast lesions and *P* values

Characteristics	Development group			Validation group		
	Benign (n=473)	Malignant (n=26)	*P*-value	Benign (n=166)	Malignant (n=15)	*P*-value
Mean age	41.85 ± 10.79	47.27 ± 10.92	<0.008*	41.72 ± 11.37	51.47 ± 13.63	0.007*
BMI	22.43 ± 2.76	22.68 ± 2.78	0.511	22.73 ± 2.76	23.58 ± 2.31	0.149
Family history			0.028*			0.031*
No	471 (99.58%)	25 (96.15%)		165 (99.40%)	14 (93.33%)	
Yes	2 (0.42%)	1 (3.85%)		1 (0.60%)	1 (6.67%)	
Age at menarche			0.606			0.349
7-11	31 (6.57%)	3 (11.54%)		11 (6.63%)	2 (13.33%)	
12-13	259 (54.87%)	13 (50.00%)		95 (57.23%)	10 (66.67%)	
≥14	183 (38.69%)	10 (38.46%)		60 (36.14%)	3 (20.00%)	
Number of births			0.396			0.058
0	74 (15.65%)	3 (11.54%)		25 (15.06%)	0 (0.00%)	
1	250 (52.85%)	17 (65.38%)		86 (51.81%)	8 (53.33%)	
2	129 (27.27%)	4 (15.38%)		47 (28.31%)	4 (26.67%)	
≥3	20 (4.23%)	2 (7.69%)		8 (4.82%)	3 (20.00%)	
Age at first birth			0.380			0.204
No birth	74 (15.64%)	3 (11.54%)		25 (15.06%)	0 (0.00%)	
<25	188 (39.75%)	10 (38.46%)		70 (42.17%)	10 (66.67%)	
≥25, <30	178 (37.63%)	13 (50.00%)		62 (37.35%)	4 (26.67%)	
≥30	33 (6.98%)	0 (0.00%)		9 (5.42%)	1 (6.67%)	
History of ADH			0.684			0.763
No	470 (99.37%)	26 (100.00%)		165 (99.40%)	15 (100.00%)	
Yes	3 (0.63%)	0 (0.00%)		1 (0.60%)	0 (0.00%)	

*P value was calculated by comparing benign and malignant lesions in pathology and indicates significant difference; BI-RADS, Breast Imaging Reporting and Data System; BMI, Body Mass Index.

**Table 3:** Univariate analysis and multivariate logistic regression analysis in the development cohort

Variables	Univariate logistic analysis		Multivariate logistic analysis	
	ORs (95% CIs)	*P*	ORs (95% CI)	*P*
**Age**	1.05(1.03, 1.08)	0.0001*	1.042 (1.00, 1.08)	0.0180*
**BMI**	1.06 (0.95, 1.18)	0.2804		
**Age at menarche**				
7-11	1.0 (Reference)			
12-13	0.55 (0.20, 1.51)	0.2439		
≥14	0.45 (0.15, 1.33)	0.1495		
**Number of births**				
0	1.0 (Reference)			
1	2.42 (0.72, 8.20)	0.1545	1.04 (0.27, 3.98)	0.9577
2	1.48 (0.39, 5.73)	0.5659	0.45 (0.09, 2.30)	0.3379
≥3	6.05 (1.36, 26.93)	0.0182*	0.92 (0.11, 7.71)	0.9374
**Family history**				
0	1.0 (Reference)			
1	10.87 (1.76, 66.98)	0.0101*	7.13 (0.62, 82.03)	0.1125
**Tissue composition**				
Heterogeneous background echotexture	1.0 (Reference)	1.0		
Homogeneous background echotexture-fibroglandular	4.09 (0.54, 30.92)	0.1723		
Homogeneous background echotexture-fat	8.49 (0.96, 74.99)	0.0543		
**Posterior features**				
No posterior features change	1.0 (Reference)			
Enhancement	0.85 (0.24, 2.97)	0.7995		
Combined pattern	2.75 (0.58, 13.05)	0.2018		
Shadowing	1.31 (0.37, 4.64)	0.6711		
**Echo pattern**				
Heterogeneous	1.0 (Reference)			
Isoechoic	0.00 (0.00, Inf)	0.9875		
Hypoechoic	0.41 (0.09, 1.92)	0.2596		
Hyperechoic	NA	NA		
Complex cystic and solid	0.71 (0.11, 4.65)	0.7173		
Anechoic	0.00 (0.00, Inf)	0.9969		
**Distance to the nipple**	1.00 (0.79, 1.27)	0.9836		
**Largest diameter**	1.25 (0.86, 1.82)	0.2489		
**Location**				
Right	1.0 (Reference)			
Left	1.07 (0.48, 2.35)	0.8749		
**Orientation**				
Not-parallel	1.0 (Reference)			
Parallel	0.44 (0.19, 1.04)	0.0610		
**Margin**				
Clear	1.0 (Reference)			
One feature	3.71 (1.73, 7.98)	0.0008	4.25 (1.56, 11.54)	0.0046*
Two or more features	11.89 (4.48, 31.56)	<0.0001*	12.30 (3.38, 44.75)	0.0001*
**Calcification**				
No calcification	1.0 (Reference)			
Coarse calcification	0.86 (0.25, 2.92)	0.8099	0.98 (0.22, 4.40)	0.9766
Micro-calcification	3.90 (1.90, 7.98)	0.0002*	4.67 (1.94, 11.26)	0.0006*
**Shape**				
Oval	1.0 (Reference)			
Round	0.00 (0.00, Inf)	0.9860	NA	0.9902
Irregular	8.28 (1.99, 34.38)	0.0036*	4.80 (0.82, 27.91)	0.0809
**Blood flow signal**				
0	1.0 (Reference)			
1	0.83 (0.17, 3.97)	0.8127		
2	1.21 (0.26, 5.54)	0.8099		
3	2.26 (0.50,10.19)	0.2876		
**Strain ratio**	1.32 (1.03, 1.69)	0.0282*	1.38 (1.04, 1.83)	0.0264*

*P value indicates significant difference. ORs: odds ratios; CIs: confidence intervals; NA: not applicable.

**Table 4:** The diagnostic performance of clinical and ultrasound nomograms in the development and validation groups

Nomogram	Group	AUC	CIs	TP	TN	FP	FN	Se	Sp	Ac	LR+	LR-	PPV	NPV
Clinical nomogram	Development group	0.6612	0.5464-0.7761	14	117	12	356	0.5385	0.7526	0.7415	2.1769	0.6132	0.1069	0.9674
	Validation group	0.7118	0.5766-0.8471	8	36	7	130	0.5333	0.7831	0.7624	2.4592	0.5959	0.1818	0.9489
Ultrasound nomogram	Development group	0.7824	0.6987-0.8661	19	144	7	329	0.7308	0.6956	0.6974	2.4004	0.3871	0.1166	0.9792
	Validation group	0.7466	0.5848-0.9084	11	48	4	118	0.7108	0.7333	0.7127	2.5361	0.3751	0.1864	0.9672

Abbreviations: AUC, area under the curve; CIs, confidence intervals; TP, true positive; TN, true negative; FP, false positive; FN, false negative; Se, sensitivity; Sp, specificity; Ac, accuracy; LR+, positive likelihood ratio; LR-, negative likelihood ratio; PPV, positive predictive values; NPV, negative predictive values.

**Table 5:** Ultrasound features of category BI-RADS 4a breast lesions and *P* values

Ultrasound features	Training group			Validation group		
	Benign (n=473)	Malignant (n=26)	*P*-value	Benign (n=166)	Malignant (n=15)	*P*-value
Distance to the nipple (cm)	2.44±1.54	2.48±1.61	0.960	2.37±1.53	3.37 ± 1.69	0.027
Largest diameter (cm)	1.47±0.89	1.68±0.76	0.052	1.50±0.87	1.59±0.69	0.225
**Location**			0.875			1.000
Right	244 (51.69%)	13 (50.00%)		75 (45.18%)	7 (46.67%)	
Left	229 (48.41%)	13 (50.00%)		91 (54.82%)	8 (53.33%)	
**Tissue composition**			0.094			0.311
Heterogeneous background echotexture	73 (15.43%)	1 (3.85%)		31 (18.67%)	4 (26.67%)	
Homogeneous background Echotexture-fibroglandular	357 (75.48%)	20 (76.92%)		126 (75.90%)	9 (60.00%)	
Homogeneous background Echotexture-fat	43 (9.09%)	5 (19.23%)		9 (5.42%)	2 (13.33%)	
**Shape**			<0.014*			NA
Irregular	6 (1.27%)	3 (11.54%)		0 (0.00%)	0 (0.00%)	
Oval	463 (97.89%)	23 (88.46%)		166 (100%)	15 (100.00%)	
Round	4 (0.85%)	0 (0.00%)		0 (0.00%)	0 (0.00%)	
**Orientation**			0.454			0.067
Not-parallel	37 (7.82%)	3 (11.54%)		16 (9.64%)	4 (26.67%)	
Parallel	436 (92.18%)	23 (88.46%)		150 (90.36%)	11 (73.33%)	
**Margin**			<0.001*			0.005*
Clear	277 (58.56%)	6 (23.08%)		106 (63.86%)	4 (26.67%)	
One feature	174 (36.79%)	14 (53.85%)		53 (31.93%)	8 (53.33%)	
Two or more features	22 (4.65%)	6 (23.08%)		7 (4.22%)	3 (20.00%)	
**Posterior features**			0.562			0.116
No posterior features change	347 (73.36%)	18 (69.23%)		117 (70.48%)	7 (46.67%)	
Enhancement	68 (14.38%)	3 (11.54%)		29 (17.47%)	5 (33.33%)	
Combined pattern	14 (2.96%)	2 (7.69%)		7 (4.22%)	0 (0.00%)	
Shadowing	44 (9.30%)	3 (11.54%)		13 (7.83%)	3 (20.00%)	
**Echo pattern**			0.559			0.006*
Heterogeneous	16 (3.38%)	2 (7.69%)		6 (3.61%)	1 (6.67%)	
Isoechoic	16 (3.38%)	0 (0.00%)		6 (3.61%)	0 (0.00%)	
Hypoechoic	407 (86.05%)	21 (80.77%)		147 (88.55%)	11 (73.33%)	
Hyperechoic	0 (0%)	0 (0.00%)		0 (0.00%)	1 (6.67%)	
Complex cystic and solid	33 (6.98%)	3 (11.54%)		7 (4.22%)	2 (13.33%)	
Anechoic	1 (0.21%)	0 (0.00%)		0 (0.00%)	0 (0.00%)	
**Calcification position**			0.028*			0.354
No calcification	374 (79.07%)	15 (57.69%)		128 (77.11%)	10 (66.67%)	
In a mass	96 (20.30%)	11 (42.31%)		38 (22.89%)	5 (33.33%)	
Outside of a mass	3 (0.63%)	0 (0%)		0	0	
**Calcification morphology**			0.003*			0.229
0	374 (79.07%)	15 (57.69%)		128 (77.11%)	10 (66.67%)	
Coarse calcification	51 (10.78%)	2 (7.69%)		19 (11.45%)	1 (6.67%)	
Micro-calcification	48 (10.15%)	9 (34.62%)		19 (11.45%)	4 (26.67%)	
**Structural distortion**			1.000			<0.001*
No	471 (99.58%)	26 (100.00%)		166 (100.00%)	12 (80.00%)	
Yes	2 (0.42%)	0 (0.00%)		0 (0.00%)	3 (20.00%)	
**Duct discharges**			0.716			0.369
No	431 (91.12%)	25 (96.15%)		148 (89.16%)	15 (100.00%)	
Yes	42 (8.88%)	1 (3.85%)		18 (10.84%)	0 (0.00%)	
**Skin Change**			1.000			1.000
Yes	5 (1.06%)	0 (0.00%)		2 (1.20%)	0 (0.00%)	
No	468 (98.94%)	26 (100.00%)		164 (98.80%)	15 (100.00%)	
**Edema**			1.000			1.000
No	472 (99.79%)	26 (100.00%)		165 (99.40%)	15 (100.00%)	
Yes	1 (0.21%)	0 (0.00%)		1 (0.60%)	0 (0.00%)	
**Blood flow sign**			0.097			0.052
3	28 (5.92%)	2 (7.69%)		13 (7.83%)	0 (0.00%)	
2	110 (23.26%)	9 (34.62%)		44 (26.51%)	8 (53.33%)	
1	167 (35.31%)	3 (11.54%)		56 (33.73%)	6 (40.00%)	
0	168 (35.52%)	12 (46.15%)		53 (31.93%)	1 (6.67%)	
**Elastography score**			<0.001*			0.141
1	35 (7.40%)	0 (0.00%)		18 (10.84%)	2 (13.33%)	
2	135 (28.54%)	1 (3.85%)		45 (27.11%)	1 (6.67%)	
3	206 (43.55%)	8 (30.77%)		73 (43.98%)	6 (40.00%)	
4	86 (18.18%)	16 (61.54%)		27 (16.27%)	6 (40.00%)	
5	11 (2.33%)	1 (3.85%)		3 (1.81%)	0 (0.00%)	
**Strain ratio**	3.47±1.38	4.08±1.20	0.010*	3.37 ± 1.34	3.46 ± 1.65	0.352

*P value was calculated by comparing benign and malignant lesions in pathology and indicates significant difference.
